# Colors in the representation of biological structures

**DOI:** 10.1515/jib-2022-0021

**Published:** 2022-07-04

**Authors:** Monica Zoppè

**Affiliations:** Institute of BioPhysics, CNR, Milano, Italy; Department of BioSciences, University of Milan, 20133 Milano, Italy

**Keywords:** color standardization, molecular animation, molecular graphics, structural biology, visualization

## Abstract

Among the many properties of proteins, sugars, nucleic acids, membranes and other cellular components, color is not present. At the same time, we humans have a natural ability of recognizing and appreciating colors, and use them generously, with the aim of both delivering information and pleasing the eyes. In this article, I suggest how we can conciliate these two situations, with the contribution of biologists, artists, and computer graphics and perception experts. The concept can be developed in a series of initiatives involving the community, including discussion sessions, technical challenges, experimental studies and outreach activities.

## 1 Introduction

Colors are among the most powerful tools in the hands of painters and illustrators. In the representation of biological structures the scientific community has used them in a variety of situations, to indicate the most disparate features of molecular properties. The results are often of stunning beauty, and indeed, the work of structural representation can be considered as much a scientific as an artistic task [1]. The number of artistically gifted scholars that help readers of scientific papers (and their less gifted colleagues) by producing figures and animations to illustrate research findings, and contribute to the dissemination of science to the public, is growing constantly, and all make use of colors. However, the color palettes employed are based on different taste, principles and logic.

In the following, I argue that, because we are so apt at seeing and noticing colors, these could be exploited in a more scientifically sound and consistent way, to indicate specific features that are relevant for the description of biomolecules, their activities, behaviours, and of their surrounding environment.

### 1.1 Colors in human culture

An preliminary note is necessary when discussing colors: in the human population, a relevant minority of male individuals have some form of Color Vision Deficiency (also known as “color blindness”), due to a series of mutations that lead to the inability to distinguish one or more colors. This fact should always be considered, as many illustrators do (but not all).

Many natural object are intrinsically colored (from sky to rocks, flowers and animal coats), and it is perfectly natural that humans have a visual system apt at seeing and recognizing them. There must have been good evolutionary reasons for the pleasure inspired by the sight of naturally colored scenes, further strengthened by millennia of cultural evolution. As we all associate emotional experiences with colors, it makes sense that our perception and interpretations are based both on the features of our physical environment as well as on cultural traditions. Inhabitants of the polar region know and interpret colors in a different way than peoples of the desert and/or of the forests, reflected in the number of words used to express different colors: while the notion that polar populations (Eskimo or Inuit) have 50 words for shades of white seems to be a metropolitan legend, the success of the fake notion testifies of how plausible we consider such an idea.

Cultural standards associated with strong emotions are perceived in an unconscious way: in a most striking example, in Europe we associate the idea of death with the color black, while in India, Japan, and other eastern cultures the corresponding color is white. If this circumstance makes it difficult to propose a color scheme that is universally interpreted, it may also mean that a new code, specifically designed for cellular and/or structural biology, could be developed independent of cultural background, thus avoiding the typical ‘cultural colonialism’ often imposed through western traditions.

### 1.2 Colors everywhere

A colored image is so much eye-catching for us, that we all are attracted and pleased when we both paint and observe them (with exceptions). Indeed, in the field of structural biology, the major graphic tools [2–4] offer the option to use ranges of colors to indicate:

– Protein, domain, and secondary structure identity
– N- to C-terminal sequence order, conservation, mutation propensity, structural stability, (un)certainty and others
– Physical properties: Electric charge, lipophilic potential, propensity for Hydrogen bonds (on aminoacids or surfaces)
– Changes in condition: ATP association/hydrolysis, activation by binding with external factors, proteolytic activation and others

Clearly, there is an interest in showing each of the mentioned features, and more, and using color is an easy and fast means to guide viewers attention, even beyond the aesthetic effect of the image as a whole.

We have to notice, however, that on one side the interpretation of such images is necessarily bound to reading the caption, and on the other that different authors use different color palettes, and even the same authors can use variable colors in different presentations.

While most of the structural illustrations published in scientific publications are made to illustrate single or a relatively small number of proteins, in the last decade, information about cellular environment can also be included in illustrations of biology: membranes, organelles, presence of Calcium, difference in pH, and other environmental properties.

A very interesting and clever development in the management of colors in dynamic multi-scale visualization was proposed by Waldin et al. [5] in which also the efficacy of the proposed scheme was assessed, even if only for the purpose of discrimination among different entities at various levels (from cellular compartment to atomic scale).

In the past, with the Scientific Visualization Group of the CNR in Italy, we have proposed a visualization method that completely avoids colors [6, 7], arguing on one side that molecules do not have intrinsic colors, and on the other that the power of colors is such that we must exert the maximum caution when using them.

However, use of B/W only is a non-solution, and the vast amount of data which can be represented, suggests a thoughtful adoption of this communication channel.

In the following I examine the varied information that can be conveyed in images, both still and animated, and suggest how a collectively agreed soft standardization may ultimately guide both illustrators and viewers interpretation.

## 2 Showing cellular and molecular biology

Cells are complex structures, that can be viewed in a range of dimensions: from the atomistic details of an enzymatic reaction to the process of chromatin condensation or of viral invasion, including all the processes at the mesoscale, in which, for example, the activation of proton pumps induces a change in pH in the lysosome, thus producing changes in conformation of proteins within the organelle. In this latter example we can illustrate the atomic details of the pump, but we can also show, in the same picture, the environments: the cytoplasm, with molecules of different size and roles, the membrane, in which are embedded many other constituents beyond the pump, and the interior of the lysosome, also populated with several proteins.

**Figure 1:**
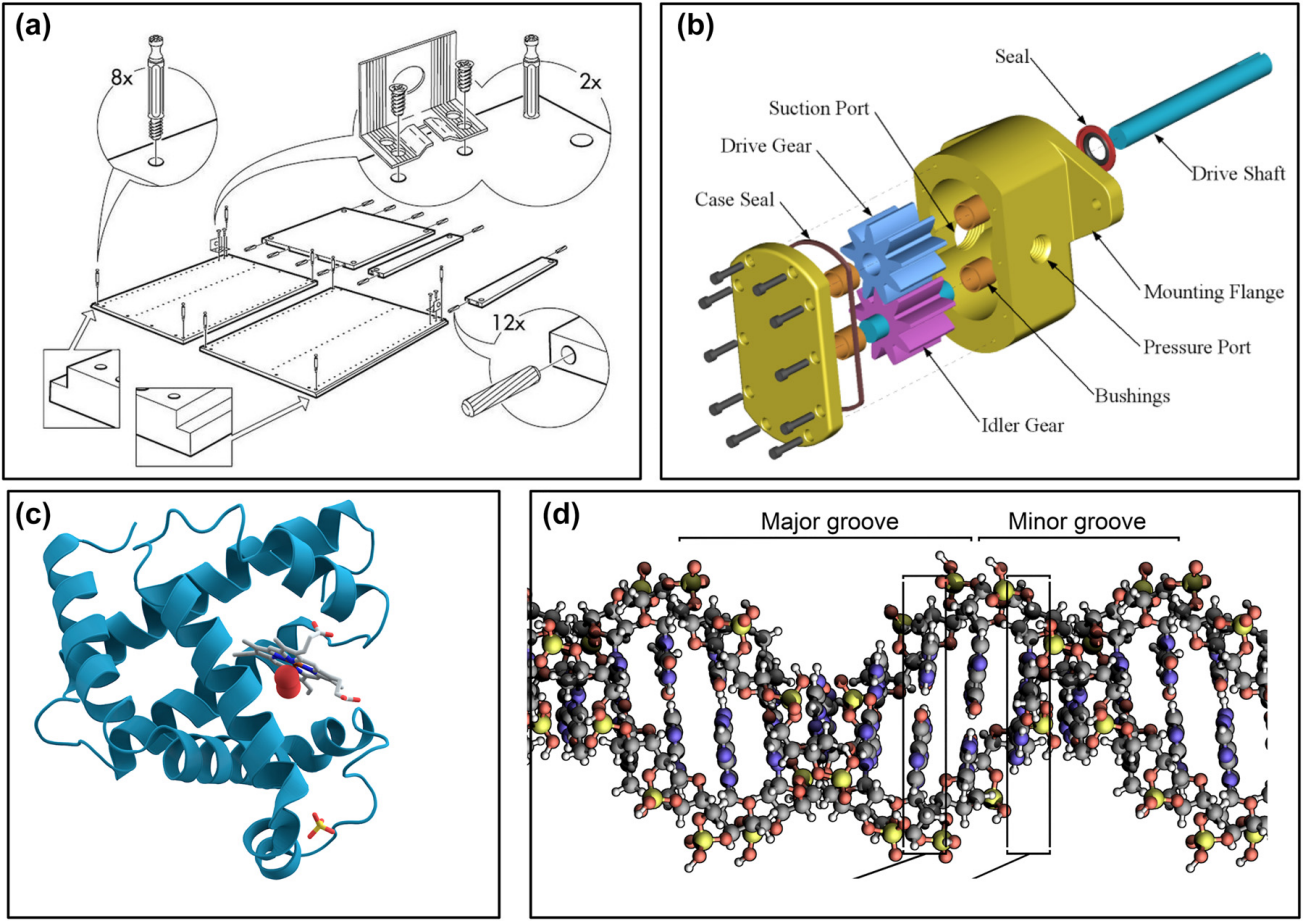
Representation of structural features can be assimilated to technical drawings. (a) Instructions for mounting furniture; (b) drawing of a gear pump [19]; (c) structure of Myoglobin [20] and (d) of DNA [21].

As mentioned, the majority of illustrations for peer communication describe a single protein, and it is supposed that viewers are familiar with the surrounding environment, or that the environment is irrelevant for illustrators and viewers. However, these are technical images, whose role is to describe specific features of a single object; as such they are more related to technical drawings than to artistic representations (Figure 1).

### 2.1 Protein–protein interactions

More and more in recent years, illustrations tend to show different proteins, their assemblies and interactions, and even complete cells [1, 8, 9]. In these cases different proteins are frequently depicted with different colors: in my opinion, supported by the experience described in Waldin et al. [5] the identity of the protein, which is determined by its aminoacid constitution, is more effectively relayed to the viewers by showing the shape, which is different for different proteins. Color could better be used to display the properties and the forces that determine the behaviour of each protein, and that are not immediately evident. We can identify and calculate at least some of these forces, but, because they are absent form our experiential (visual) world, we do not have an intuitive immediate way to represent them. Here, colors can be exploited, i.e. to introduce concepts that, because they are invisible to us, can be represented with arbitrary principles.

It is important to show them, because, in cells, besides its shape and intrinsic motions, what determines the activity of each protein is the relation that it entertains with other proteins and molecules, which is in turn determined by the forces acting in the protein space. Electrical, hydrophobic, Van der Waals and H-bonding forces, i.e. weak local chemical interactions, are important features, and it does make sense to show them, with colors or other visual clues. This will add informative dimensions to our capacity of reading images of cellular scenes. The idea, of course, is not new: most visualization programs can show these features, but hardly simultaneously, certainly not in intuitive way.

*Ionic interactions.* The “standard coloring” of electric charge for biologist (negative red and positive blue, associated with Oxygen and Nitrogen, respectively) is inverted relative the conventional colors used in physics and engineering (negative red and positive blue or black). However, even knowing the charge polarity, and even after many years using the convention, it is not immediately evident that opposite charges (colors) attract and equal charges repel each other. My suggestion would be that charge is represented by particles, possibly directional, like small comets or arrows, of a single color, flowing in the direction (again, as a convention widely accepted) positive to negative, along the field lines associated with the local charges.

**Figure 2:**
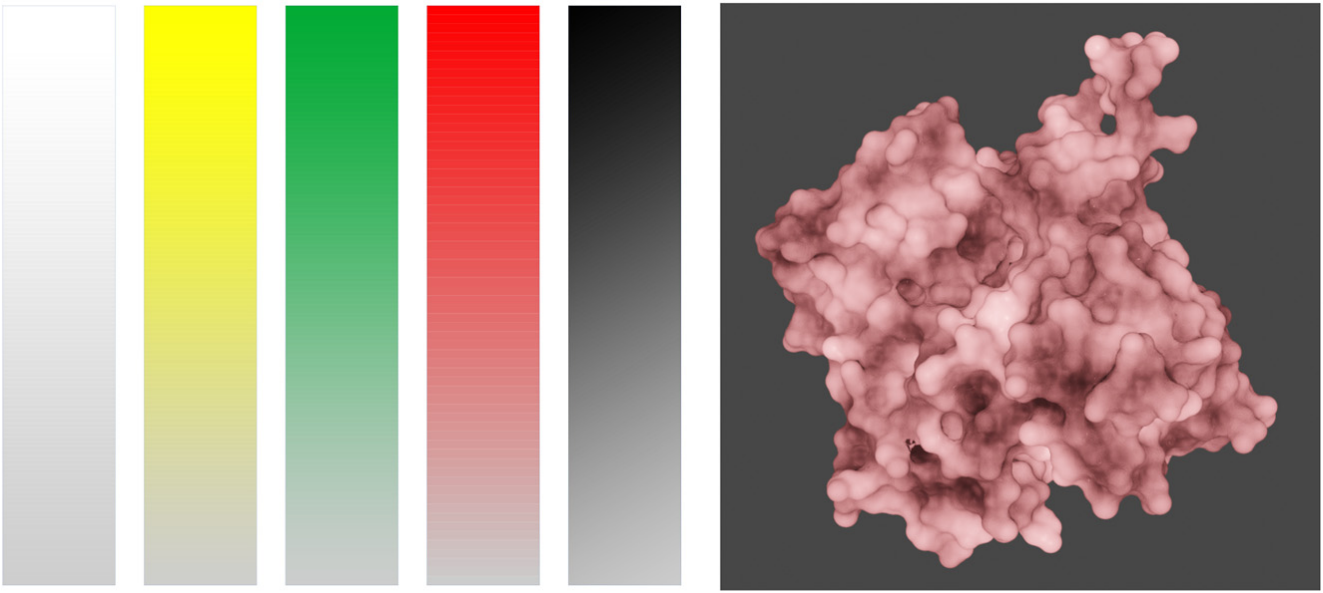
Any color can have a range of intensity (**left**) that can be used to convey properties expressed with a continuum (**right**). Actin monomer (from PDB entry 6anu, B) displaying lipophilic potential calculated and rendered using BioBlender [22].

*Hydro-philic and -phobic potential.* This is another feature frequently available from visualizations tools, and frequently expressed in colors. Because this property can be expressed as a continuum (depending on how it is calculated), grades of saturation or intensity, applied to any color of choice (even white) could convey the information (Figure 2). In this case, the effects of surface brightness, shadows, occlusion, and other aspects of illumination must be considered so that the resulting image relays information and is not confusing. Other solutions could be explored, such as the use of graded texture of reflections.

*Hydrogen bond propensity or polarity*. Not as easily represented as the two properties above, but relevant in protein–protein and protein–ligand interactions, the H-bond propensity could be represented with short filaments projecting from the relevant atoms, in particular as Hydrogen atoms are rarely shown, and are often excluded in the calculation of the protein surface. This would reveal interactions with both water (usually not shown) and with other molecules. Since also this force is directional, some visual clue to indicate polarity should be introduced.

For the three forces above, the representation proposed would also indicate the spatial range of their effect, with electric forces projecting up to several Å from the molecular surface, H-bonds in the order of about 2 Å, and hydrophobic forces working practically at the surface level.

### 2.2 Cellular environments

Proteins do not live in the vacuum; they spend their lives, from synthesis to degradation in one or more different cellular environment. The environment of the cytoplasm is different from the environment in the nucleus or in other places, and from the extracellular space.

In our real world, the picture of a subject is always inserted in a background: for human subjects it could be a sofa, a beach, a mountain, a street… We recognize these environments because they have typical features.

Cellular biology gives us indications for “setting the scene” and populating the different environments: ribosomes and cytoskeletal elements in the cytoplasm; narrow spaces in the ER and Golgi; walls decorated with electron transport elements for mitochondria and for chloroplasts (in this latter case with the green chlorofill antennae); DNA in the nucleus, organized in eu- and hetero-chromatin in different zones within it; structural networks and extracellular matrix on the two sides of the plasma membrane and so on.

In theory, therefore, we do not need to use colors to distinguish among places. Nevertheless, it might be possible to consider a “colored light”, that illuminating the scene, confers to the different places a typical tone, thus helping in their recognition. Colored light is an easy tool to use in 3D scenes, and interferes only slightly with the colors of other objects in the scene, if any. The added information, if used consistently, would help viewers to locate the protein.

**Figure 3:**
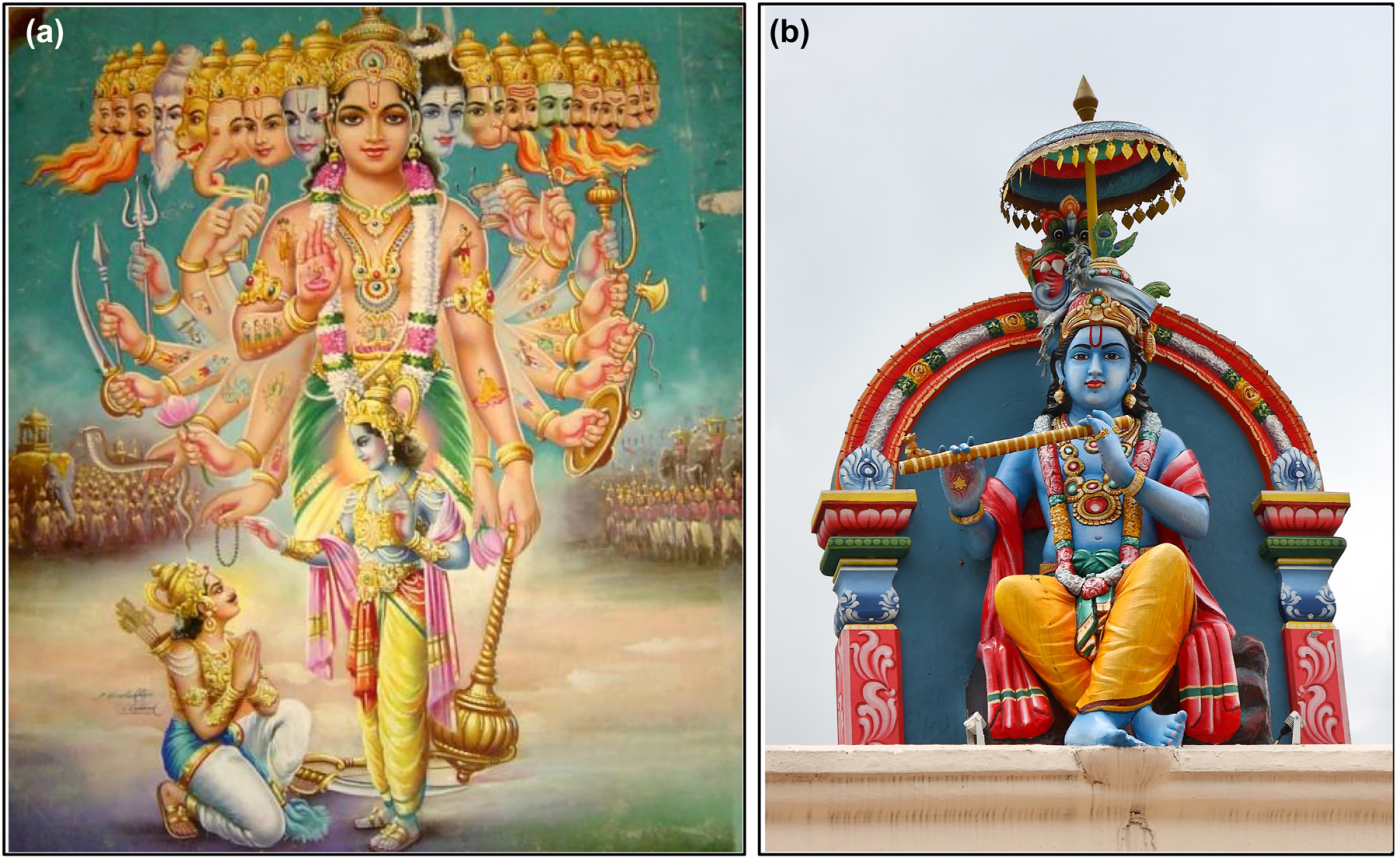
2D [23] (a) and 3D [24] (b) representations of Krishna. In (a), it is represented the episode of Krishna proving prince Arjuna with help, advice and powers for the preparation of a battle (armies in the background).

**Figure 4:**
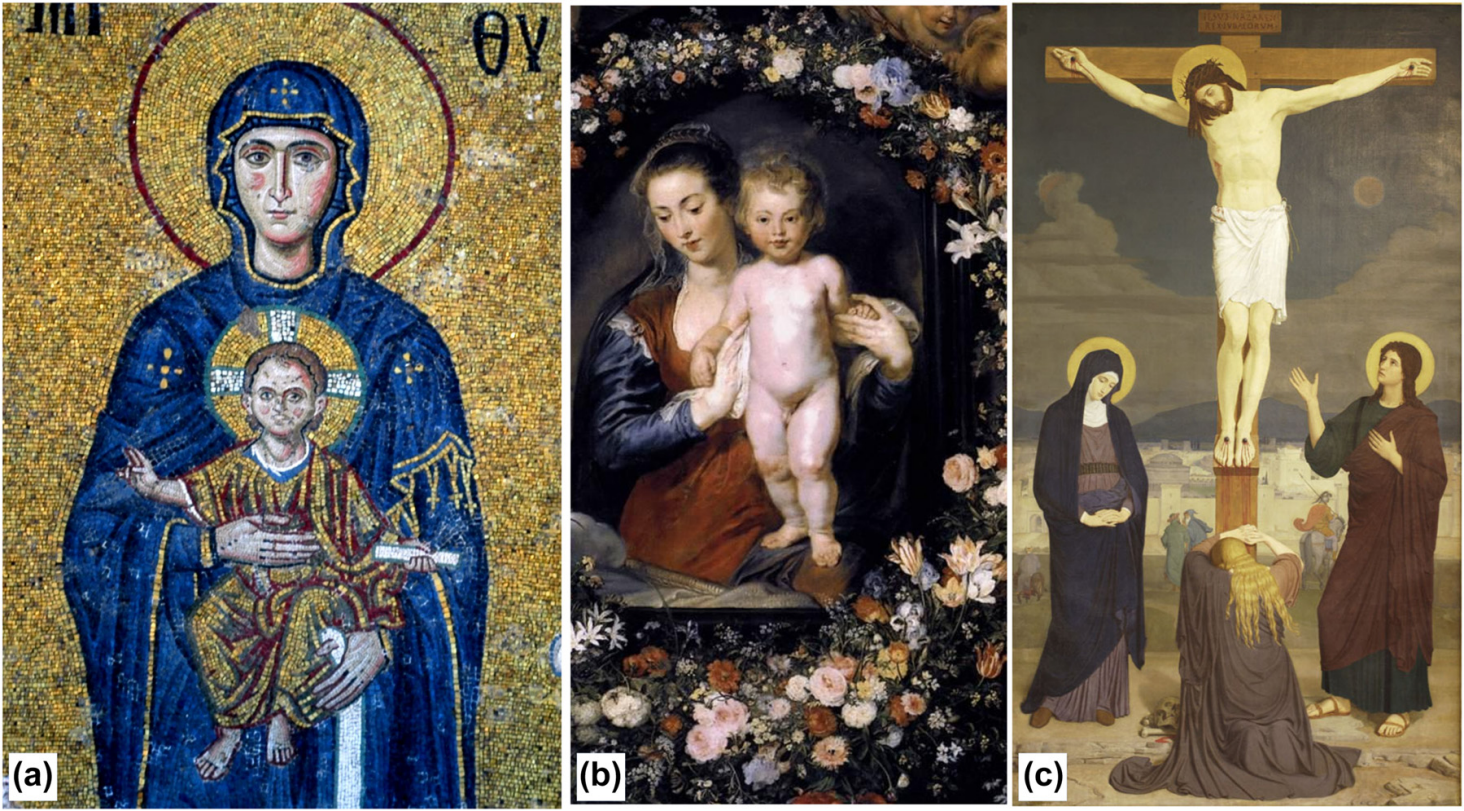
Representation of the Virgin Mary with the baby (a and b), [25, 26] and in the Stabat Mater (c, [27]). In all images Mary is dressed with blue colors, which make her immediately recognizable, also among the other figures (Magdalena and St. John) under the cross in (c).

Painters in the past centuries have used colors to indicate the identity of specific characters, making them recognizable in many different circumstances. For example, Krishna is typically depicted with blue skin, and people have assimilated in their culture this property, so that the God is recognized in whatever place (Figure 3(a) and (b)). Similarly, the Virgin Mary has almost invariably been represented dressed in blue, which makes it easy to identify her with the baby (Figure 4(a) and (b)), or under the cross where other subjects are present (Figure 4(c)). This color standardization may have originated in relation to symbolic meaning attributed to specific colors, or to other factors; in any case, it has not limited artistic freedom, as the very different images of Figures 3 and 4 demonstrate.

### 2.3 Other principles of art design as applied to cell biology

Sometimes colors are used to highlight specific features of a protein or of a complex. But colors are only one of the many tools in the hands of illustrators: lighting and composition are extremely powerful means to direct viewer attention to specific details, and lines can be embedded to indicate sequential steps of a process. While illustrators have made very liberal use of colors, these other principles of art design have not be exploited as much. One reason might be the desire to stay faithful to the images obtained by direct observation of cells and tissues using microscopy, or to represent the exceptional crowdedness of cellular environments. However, just like we feel authorized to use colors where none exists, we can also take the liberty of reducing the crowd, for the sake of clarity. Additionally, sound effects can also be considered.

### 2.4 For a culture of cellular biology

The reason why we all recognize iconic figures of our culture is because they are embedded in our environment, we see and hear about them from the early age, so that they have become familiar. We also identify places where we have never been, and people we have never met.

The same cannot be said (yet?) for cellular biology: the first place where cells are presented to us is at school, but this is also true for Tutankhamon and Julius Caesar (at least in Italian schools). The difference is that Egyptian and Roman kings and emperors have a stereotyped iconography, presented in consistent way, even when simplified to the extreme for the smallest kids. For inanimated objects, the same can be said about the pyramids, the Colosseum, the Great Wall of China and the Taj Mahal: almost no kid has seen them in person, but they do know and recognize them when depicted in drawing or photograph. It helps that they are also connected with stories.

A general outline for representing stereotyped cells is available, also for young children [10] coloring books. I think that we could introduce colors with an agreed meaning such that it can be used for scholars to communicate the molecular details and for kids to get general ideas (at first) about cells and their constituents.

## 3 Coloring biology

Cells are colorless, and the cellular world is organized following chemical and physical principles that we cannot see (including time, here not discussed). In our world, and with our visual system, we are unable to see cells directly, but we use many techniques to obtain information on the cellular world. The challenge of representing them has been faced and solved with many creative tools for about 60 year, from cardboard models [11] to virtual reality [12]. However, most of these efforts have been made in an uncoordinated way, and we have now a range of visualization methods and styles.

The challenge here is to put together scientific rigour and artistic freedom, where the former is supposed to be universally fixed and the latter totally arbitrary.

Even in this difficult situation, however, some “constants” can be suggested, and I think that if we can accept them, it would be beneficial for those who want to represent some biology, but are not artistically gifted, for those who are, (for whom is valid the principle expressed by cartoonist Jules Feiffer that “Artists can color the sky red because they know it is blue.”), and for viewers, that is, almost everyone.

Sometimes standards develop “spontaneously”, due to the success of a new concept or word: for example, we all use the word ‘scotch’ to indicate the adhesive tape, and, in the field of structural biology we all use and easily interpret the ribbon style of protein skeleton description [13]. In other cases, they are the result of discussions among experts, and this is the case for many scientific definitions, regulated by committees, such as IUPAC (the International Union of Pure and Applied Chemistry, ref. [14], ICZN (International Commission on Zoological Nomenclature, ref. [15]), and many others.

These committees arose following periods of spontaneous and creative confusion, with the aim of making it easier to communicate and understand each other. In the field of visualization and structural biology, in 1987 a panel was convened to discuss Visualization in Scientific Computing [16]; more recently, a focused discussion took place in 2018 at the Shonan Meeting on Web Molecular Graphics [17].

I suggest that the time has come to gather the many practitioners of molecular and cellular visualization, to discuss and possibly establish a few principles about colors that may develop into standards.

### 3.1 Framework for a discussion

The first issue to be discussed is about the opportunity of establishing a standardization on the use of colors in molecular graphics: this can be initially discussed at the meeting, and possibly further explored through an online survey in the community. If there is general agreement on this fundamental point, the best option for such a discussion is to organize one or more workshop (possibly in person) with the contribution of structural and cell biologists, experts of human visual perception, developers of the graphic tools, and, of course, the major practitioners of our days.

While in most of the examples considered, especially for large, mesoscale visualizations, color is used to discriminate between different components, some of the discussion should also focus on if and how to represent forces and relationships. The limited suggestions made in this article can serve as a discussion basis, not assuming that they are the most convenient, considering the various scientific, artistic and technical aspects.

Technical aspects have also to be considered, in particular in relation the means of delivery (printed on paper, video/film, interactive video, even physical models [18] and Virtual reality [12]). This is likely to require detailed discussions, depending on what precisely we will aim at showing, and how it is defined or calculated.

Once agreed among the necessarily limited number of components for this (hypothetical) committee, the proposed standards should be published on major journals and widely distributed, providing space for further discussion, before being fixed (temporarily). The efficacy of such choices could be tested in experiments involving schools up to the university level (students and teachers), general public, interested public (like patients organizations), and scientists.

Implementation could be achieved by requesting the developers of CG to default selected option, and warn about changing them. Editors could help requesting that images in the scientific literature are made in agreement with the standards, unless specific reasons advise otherwise.

I am looking forward to the possibility of discussing the issue with esteemed colleagues worldwide, and would be ready to contribute in preparing a proposal to request funding for such multidisciplinary project.
